# The effect of whole body vibration training on quadriceps voluntary activation level of people with age-related muscle loss (sarcopenia): a randomized pilot study

**DOI:** 10.1186/s12877-018-0923-z

**Published:** 2018-10-11

**Authors:** Ning Wei, Gabriel Y. F. Ng

**Affiliations:** 1 Department of Rehabilitation Sciences, College of health science & Nursing, Wuhan Polytechnic University, Room309, Hubei, China; 2 Department of Rehabilitation Sciences, The Hong Kong Polytechnic University, ST828, Hung Hom, Hong Kong SAR; 3 Department of Rehabilitation Sciences, The Hong Kong Polytechnic University, QT510a, Hung Hom, Hong Kong SAR

**Keywords:** Age-related muscle loss, Whole-body vibration, Voluntary activation, Twitch interpolation

## Abstract

**Background:**

Whole body vibration was an effective training for improving muscle performance. The purpose of this study was to explore the effects of 12-week whole-body vibration training program on voluntary activation of quadriceps muscles of older people with age-related muscle loss (sarcopenia).

**Methods:**

Twelve community dwelling seniors with age-related muscle loss were randomly allocated into whole body vibration training group and control group. The training lasted for 12 weeks. Twitch interpolation were conducted to examine the voluntary activation of quadriceps at pre- and post-intervention.

**Results:**

Although there was no significant difference between whole body vibration training group and control group on the absolute values of the interpolated twitch ratio after 12 weeks of training. The changed values of ratio (Post minus Pre) were significantly different between the two groups (*p* = 0.044).

**Conclusions:**

The voluntary activation of quadriceps muscles of older people with age-related muscle loss was facilitated after 12 weeks of WBV training with 40 Hz × 4 mm × 360 s. Considering the small sample size of this study, it may only provide a piece of evidence that WBV is effective for facilitating the central motor drive in seniors with age-related muscle loss. More subjects are needed to confirm the present finding.

**Trial registration:**

ISRCTN63583948, registered on 16th January 2017, retrospectively registered.

## Background

The age-related muscle loss (sarcopenia) is accompanied by decreased muscle strength, which would lead to the disability and failure in physical performance in daily life. Loss of motor neuron, increased nerve to muscle innervation ratio and remodeled motor unit were the main contributors to age-related loss of the muscle mass and impairment of force production [1–3]. It is known that suitable type and sufficient level of exercise training would benefit the muscle mass and strength in man [4–6]. However, many studies had reported that the increase in muscle strength was not in line with the changes in muscle mass [7–9]. Considering the non-parallel relationship between the training-induced changes in muscle mass and strength, neural adaptation would be a contributor to the increased muscle strength in the early phase of training.

Some previous studies confirmed that the voluntary activation of muscles was facilitated after long-term physical training [10, 11]. Scaglioni et al. examined the voluntary activation of plantar flexor of 14 healthy older adults and found the voluntary activation significantly increased after 16 weeks of strength training [10]. Knight and Kamen reported the voluntary activation of knee extensors increased in both young and old subjects after 6 weeks of resistance training [11].

As a popular physical training, whole body vibrating training could improve the performance of muscle strength in both young and old subjects [12, 13]. Delecluse et al. found the peak torque of isometric and isokinetic knee extension had increased by 16.6% and 9%, respectively, in young adults after 36 sessions of WBV [12]. One recent study reported that more than 15% increase in isometric knee extension peak torque was presented in the older people with age-related muscle loss (sarcopenia) after completing a 12-week WBV training program [13].

The tonic vibration reflex has been suggested as a plausible underlying mechanism that explains the benefits of WBV training [14]. The vibration stimulates muscle spindle discharges, which activate the monosynaptic and polysynaptic reflex arcs through the afferent nerve fibers causing muscle contraction. Besides TVR, neural adaption was speculated as another possible underlying mechanism of WBV training [14, 15]. Until now, there was no study examining neural adaption after long-term WBV training. The contribution of neural adaptation to the improvement of muscle performance was still unclear. Therefore, the aim of this pilot study was to investigate the effect of 12 weeks of WBV training on the voluntary activation of quadriceps muscles in older people with age-related muscle loss.

## Methods

Subjects aged 65 years or above with no uncontrolled medical conditions attending the local Elderly Health Centers were invited to go through a non-invasive screening test of bioelectrical impedance measurement so as to estimate their absolute skeletal mass. An established formula [16, 17] was used to calculate the skeletal mass that:

$$ Skeletal\ mass=\left[{0.401}^{\ast }\ \left({height}^2/ bio- impedance\right)+\left({3.825}^{\ast }\  gender\ index\right)-\left({0.071}^{\ast }\  age\right)+5.102\right]. $$

$$ Gender\ index\ for\ male=1; female=0. $$

The absolute skeletal mass was converted to skeletal mass index by dividing it with the square of body height. Male and female participants with skeletal mass index less than 8.87 kg/m^2^ and 6.42 kg/m^2^, respectively, would be classified as sarcopenic [16, 18] and invited to participate in this study. Subjects with metal implants, severe heart problem, neurodegenerative diseases, peripheral vascular diseases, vestibular disorders, severe osteoporosis or fractures to the weight bearing bones within one year prior to the study were excluded. The exclusion criteria were based on the contra-indications for whole body vibration training in the previous studies [4, 19].

**Fig. 1:**
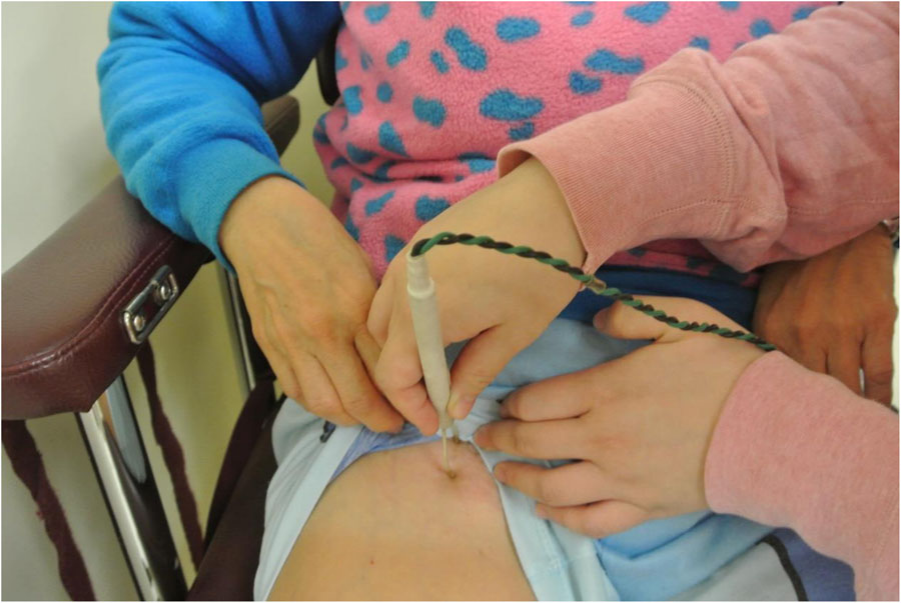
The participants undertaking twitch interpolation test. The stimulating electrode was placed over the femoral nerve trunk underneath the femoral triangle

This study adheres to CONSORT guidelines. The recruited subjects were equally randomized into WBV training group (WBV) and control group (CON) by a researcher with a computer program (Research Randomizer Form www.randomizer.org/integer-sets/). Each participating subject gave voluntary informed written consent before participating the study. The consent was obtained from the subjects before using their images in this article (Fig. 1). The procedures were reviewed and approved by the Human Ethics Review Board of Department of Rehabilitation Sciences of Hong Kong polytechnic University prior to commencement of the study. The clinical registration number is ISRCTN63583948 on 16th January 2017.

All training sessions were conducted in a sports training laboratory of the administrating institution under the supervision of a physical therapist. A total of 36 training sessions were implemented at 3 days/week over a 12-week period. There was a minimum rest of one day between the training sessions. Extra sessions catering for missing appointments were arranged to make sure all participants would complete all sessions.

In this study, the parameters of WBV training was set according to our previous study, which revealed that the optimal combination of frequency and exposure time per set was 40 Hz and 90 s [13]. The training on each day comprised four sets with each containing 3600 vibration cycles. The peak-to-peak amplitude was set at 4 mm.

During training, the participants stood barefoot with knee joint flexed at 60° on the platform of a whole body vibration machine (Fitvibe excel, GymnaUniphy NV, Bilzen, Belgium) and hands holding onto the rail in front for support. A soft mat supplied by the Fitvibe manufacturer was placed on the vibration platform during all training sessions for protection. Participants were advised to keep their lifestyle and physical activity as usual during the study period.

All participants were assessed at baseline and post-intervention (12 week) by a professional researcher who was blinded to the intervention. The neuromotor recruitment of the quadriceps muscle was tested using the twitch interpolation technique. Twitch interpolation is a technique that involves electrical stimulation to the muscle nerve with a single pulse during a maximal voluntary contraction to elicit an increment of force [20]. It is a noninvasive technique detecting the training-induced changes in voluntary activity. Considering the physical conditions of the participants, twitch interpolation is relatively safe and simple with the logistics.

The test was conducted in each group before and after 12 weeks of training to measure the changes in motor unit activation in the quadriceps of the dominant leg [21]. Participants sat on an isokinetic testing chair with hip at 80° and knee at 90° of flexion and performed a maximal isometric knee extension. The knee extension force was simultaneously shown on a digital indicator (AD-4532A, A&D company, Tokyo, Japan) and converted to digital output by a 12-bit analog-to-digital converter (NI USB-6211, National Instruments, Austin, USA) with sampling frequency at 1 kHz. A custom-made stimulation electrode connected to an electrical stimulator (S88 Square Pulse Stimulator, Grass Technologies, Warwick, USA) was placed on the femoral nerve trunk after the skin was cleansed with exfoliating scrub (Fig. 1). The location of femoral artery was firstly determined by palpation within the inguinal triangle. The pulse from the femoral artery was detected and this was used as the landmark to locate the femoral nerve, which sits immediately lateral to the femoral artery. Then several submaximal electrical stimulations were delivered along the course of the femoral nerve trunk to search the best location of applying the stimulation pulse and determine the supermaximal electrical stimulation that the subjects could tolerate.

Participants were asked to perform 3 maximum voluntary extensions (MVCs), which lasted for 2–3 s. When the peak force was obtained, a supramaximal electrical stimulation was delivered to the femoral nerve trunk to trigger a superimposed quadriceps muscle twitch. Afterwards, the participant would relax the quadriceps for 5 s and another supramaximal electrical stimulation was applied to the femoral nerve trunk to elicit a control muscle twitch of the quadriceps. The data were stored for calculating the interpolated twitch ratio.

Two sessions of measurements were conducted on two separate days to examine the test-retest reliability of twitch interpolation. The interval of the testing sessions was 7 days apart and in order to avoid the circadian effect, the tests were conducted in the same hour of the two days. Twitch interpolation showed good test-retest reliability (ICC_3,1_ = 0.811).

Descriptive analyses were reported as means ± standard deviations. Shapiro-Wilk test was used to examine whether the data followed normal distribution. To compare the baseline characteristics of the two groups, independent-sample t-test was conducted. The changes of interpolated twitch ratio were calculated as Post minus Pre in both groups. Independent-sample t-test was performed to compare the between-group difference in the changes of interpolated twitch ratio. Paired t-test was used to investigate the within-group changes. Intention-to-treat (ITT) analysis was used. The last observation carried forward method (LOCF) was used to handle missing data due to attrition. SPSS 20.0 (SPSS Inc., Chicago, Illinois, USA) was used for statistical analysis. Significance level was set at *p* < 0.05, unless otherwise stated.

## Results

**Fig. 2:**
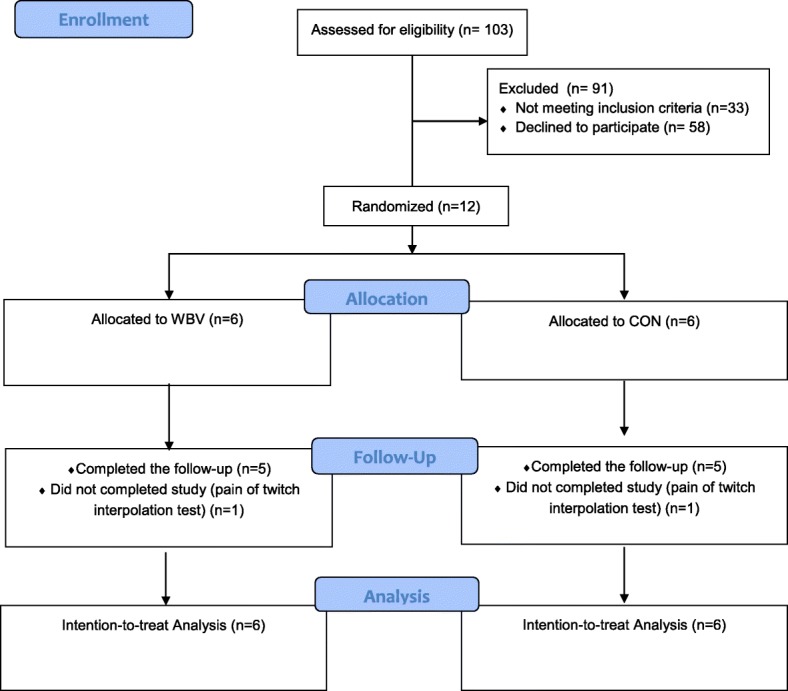
Flowchart of group assignment for the study. WBV: whole body vibration group; CON: control group

Twelve subjects (3 males and 9 females, aged 74.3 ± 5.2 years) were recruited to this study. Two subjects withdrew due to the pain of twitch interpolation test. Finally ten subjects completed this study (Fig. 2). Baseline characteristics of participants were summarized in Table 1. There was no significant difference between groups in physical characteristics and interpolated twitch ratio at baseline (*p* > 0.05).

**Table 1:** The characteristics of participants at baseline assessment (Mean(SD))

	WBV (*n* = 6)	CON (*n* = 6)	*p* value
Age (yrs)	73.6 (4)	74.8(6)	0.717
Height (cm)	152.5(7.4)	156.6(5.4)	0.293
Weight (kg)	52.35(5.8)	53.3(4.5)	0.770
BMI (kg/m^2^)	22.65(3.32)	21.71(1.69)	0.549
SMI (kg/m^2^)	6.54(1.13)	6.31(1.19)	0.728

WBV: whole body vibration group; CON: control group; BMI: body mass index; SMI: skeletal mass index. The p values were for between group comparisons

Neither within- nor between-group changes on the absolute values of the interpolated twitch ratio were significant after 12 weeks of training (p > 0.05). However, the changed values of ratio were significantly different between the two groups (*p* = 0.044; 95%CI: 0.089, 5.569) (Table 2).

**Table 2:** The effects of a 12-week WBV training on interpolated twitch ratio (Mean(SD))

	WBV (*n* = 6)	CON (*n* = 6)	*p* value
Pre	71.92%(8.38%)	74.20%(4.73%)	0.575
Post	73.00%(8.58%)	72.48%(4.62%)	0.892
Delta	1.11%(2.48%)	−1.72%(− 1.71%)	0.044

WBV: whole body vibration group; CON: control group; Pre: before training; Post: after completion of 36 training sessions; Delta: the changes of interpolated twitch ratio between before and after training; BMI: body mass index; SMI: skeletal mass index. The *p* values were for between group comparisons

## Discussion

In this study, the interpolated twitch ratio increased from 71.92 to 73.0% in the subjects with a 12-week WBV training program, while those in the control group showed a decrease by 1.72%. Since no previous study had investigated the interpolated twitch ratio after WBV training in older people with age-related muscle loss, this is the first study to explore the WBV-induced changes in the levels of voluntary activation in older people with age-related muscle loss (sarcopenia).

According to the literature, only one study had applied the twitch interpolation technique to investigate the effect of a 6-week WBV training program on voluntary activation [22]. They found voluntary activation of knee extensors did not change after 6 weeks of WBV training program. However, the subjects participated in that study were active college students, who already had high level of voluntary activation before training. In the present study, the subjects were older people with age-related muscle loss, who had lower level of voluntary muscle activation than their young and healthy counterparts [3]. Therefore, the different findings between the present study and that of Petit et al. are not surprising due to the different characteristics of subjects.

One possible mechanism of the WBV training effect is due to TVR. Some studies had proven the existence of TVR during WBV vibration [23, 24]. Zaidell et al. reported EMG amplitudes of soleus and tibialis anterior increased during WBV training [23]. Shinohara et al. examined the maximal motor unit discharge rate of the first dorsal interosseous muscle in 32 young adults after 30 min of vibration training with 75 Hz and reported the discharge rate to have increased after vibration training [24]. Furthermore, they also found that the amplitude of the short-latency component of the stretch reflex increased after vibration. The WBV movements stimulate the sensory input of muscles with external vibration, which would increase the excitatory input to α motor neurons though Ia afferents in muscle spindle [24]. Since Ia afferents from muscle spindle is sensitive to the changes of muscle length, vibration would activate the homonymous motor units, which induce the muscle strength increase [25].

However, TVR might not be the only underlying mechanism of WBV training. Considering WBV training is a form of exercise training such that neural adaptations would also occur after long-term of WBV training. Roll et al. and Ushiyama et al. found the H-reflex amplitude would decrease after prolonged vibration [26, 27]. The H-reflex is usually used for assessing the excitability of motor neurons, which in turn determines the motor unit activation [28]. The changes in H-reflex after vibration hinted that there could be some other possible underlying mechanisms of the effects of WBV.

In the present study, the increase of the twitch interpolation ratio in the quadriceps after 12 weeks of WBV training indicated WBV training could facilitate the central motor unit drive during voluntary muscle contraction. This study has provided the first piece of evidence that long-term WBV training is effective on facilitating central drive. Many studies had proved the fact that long-term training could improve the voluntary activation of muscles [10, 11].

While some studies applied the twitch interpolation technique and reported resistance training could not induce any increase in voluntary activation in healthy young and old subjects [29, 30]. The explanations for the divergent findings might be due to the difference in the subjects and training protocols in the studies. Herbert et al. reported the voluntary activation of 44 college students had only marginally increased from 96.2 to 96.9% after 8 weeks of resistance training [29]. Since those subjects were active young people, the voluntary activation of their muscles was already at a high level before training, thus they would have limited capacity for further improvement. Sale et al. trained their subjects with dynamic exercise and found no change in isometric strength and voluntary activation [30]. Considering the principle of specificity with exercise training, it is unreasonable to expect large increase in isometric strength with dynamic training program.

There are some limitations in this pilot study. First, the sample size of this pilot study is relatively small. As discussed in the method session, the twitch interpolation test is uncomfortable for the participants thus it was difficult to recruit subjects to participate in this study. The twitch interpolation test was only completed on 5 participants in each group. In order to eliminate some bias due to the dropouts, an intention-to-treat (ITT) analysis was used for data analysis. Study with large sample size is needed to confirm this present finding in the future. Second, the definition of sarcopenia might be inaccurate in this study according to the study of Cruz-Jentoft et al. (2010). However, as we planned our huge project, including this pilot study, there was no consensus on the definition of sarcopenia at that time. Thus, we applied the definition of sarcopenia in the study of Chien et al. (2008) to this study. Third, bio-impedance measurement was conducted without overnight fasting. However, considering the health condition of our targeted population, it is dangerous for them to fast overnight. Forth, the possible effect of antagonists and synergists on the peak force had not been taken into consideration. However, since the interpolated twitch ratio is a relative value, if there is a systemic effect between the antagonists and synergists, it should have happened in each trial thus the effect of which would be eliminated and not reflected in the value of the interpolated twitch ratio. Fifth, there was no placebo group in this study. The changes of voluntary activation may not only due to vibration therapy, but also static squatting on vibration platform. It would have been better to have a placebo group that only received static squatting training without vibration.

## Conclusion

The voluntary activation of quadriceps muscles of older people with age-related muscle loss was facilitated after 36 sessions of WBV training with 40 Hz × 4 mm × 360 s of training dosage per session over a 12-week period. This provides the first piece of evidence that WBV is effective for facilitating the central motor drive in seniors with age-related muscle loss (sarcopenia). Based on the present findings, it is suggestive that the future study for investigating the possible mechanism of WBV training should target on the contractility and muscle synergies rather than the facilitation of the central motor drive.

## Abbreviations

TRV: tonic reflex vibration
WBV: whole body vibration
